# ZnO Nanoparticles: A Promising Anticancer Agent

**DOI:** 10.5772/63437

**Published:** 2016-01-01

**Authors:** Gunjan Bisht, Sagar Rayamajhi

**Affiliations:** 1 Department of Chemical Science and Engineering, Kathmandu University Dhulikhel, Nepal; 2 Department of Biotechnology, Kathmandu University Dhulikhel, Nepal

**Keywords:** Zinc Oxide (Zno), Anti-cancer Agent, Reactive Oxygen Species (ROS), Selectivity, Cytotoxicity, Zinc-mediated Protein Activity Disequilibrium, Enhanced Permeation and Retention Effect (EPR)

## Abstract

Nanoparticles, with their selective targeting capabilities and superior efficacy, are becoming increasingly important in modern cancer therapy and starting to overshadow traditional cancer therapies such as chemotherapy radiation and surgery. ZnO nanoparticles, with their unique properties such as biocompatibility, high selectivity, enhanced cytotoxicity and easy synthesis, may be a promising anticancer agent. Zinc, as one of the major trace elements of the human body and co-factor of more than 300 mammalian enzymes, plays an important role in maintaining crucial cellular processes including oxidative stress, DNA replication, DNA repair, cell cycle progression and apoptosis. Thus, it is evident that an alteration in zinc levels in cancer cells can cause a deleterious effect. Research has shown that low zinc concentration in cells leads to the initiation and progression of cancer and high zinc concentration shows toxic effects. Zinc-mediated protein activity disequilibrium and oxidative stress through reactive oxygen species (ROS) may be the probable mechanism of this cytotoxic effect. The selective localization of ZnO nanoparticles towards cancer cells due to enhanced permeability and retention (EPR) effect and electrostatic interaction and selective cytotoxicity due to increased ROS present in cancer cells show that ZnO nanoparticles can selectively target and kill cancer cells, making them a promising anticancer agent.

## 1. Introduction

Cancer, a condition of uncontrolled cell differentiation, has usually been treated by chemotherapy, radiation and surgery during the past several decades [1]. These therapies are certainly efficacious in the destruction of cancer cells, but, alongside that, they come with the cost of an increasing rate of adverse consequences due to unselective effects directed towards normal cells as well [2]. These therapies are now gradually becoming outdated in cancer treatment due to the development of nanomedicine, targeted drug delivery and multi-target inhibitors [3]. Nanomedicine is the field of biomedical application of nanotechnology in which engineered nanoparticles (NPs) are used to treat disease. Nanomedicine, with its advanced imaging and therapeutic capabilities, has the potential for early detection of cancer and cancer treatment [4]. It has the additional benefits of active/passive targeting, high solubility/bioavailability, biocompatibility and multifunctionality over traditional cancer therapies [5].

Nanomaterials show the following unique properties, due to which they have recently become a widely discussed research topic and a preferable substitute to conventional cancer treatment methodology: 1) Biomolecules, whose size is comparable with nanoparticles, play an important role in regulating various cellular cycles of the body and maintaining crucial cellular homoeostasis. With proper engineering, NPs can be localized in any system of the body and mimic the activity of biomolecules, thus hacking the system biology of the body according to the need for human benefit. 2) NPs are highly soluble due to their small size and their solubility can be further increased by proper surface modification. 3) Due to NPs' high surface area to volume ratio, they have ample surface area to encapsulate drugs and other materials, thus providing higher therapeutic payload. 4) Due to their selective targeting nature, NPs can specifically release a therapeutic payload onto the target, reducing the side effects on normal cells [4, 5]. Besides cancer, nanomedicine is now showing increasing application in personalized medicine [6] and diagnosis and therapy of cardiovascular diseases [7].

The major aspect of nanomedicine comprises inorganic NPs. Many inorganic NPs conjugated with anti-cancerous drugs or bio-active molecules (peptides, proteins, DNA, etc.) have already been approved by the U.S. Food and Drug Administration (FDA) and European markets, such as Feridex, Resovist, Doxil, Abraxane, etc. [4]. Furthermore, inorganic NPs themselves show selective cytotoxicity towards cancer cells [8]. Inorganic NPs such as iron oxide NPs, titanium dioxide NPs, cerium oxide NPs, zinc oxide NPs, copper oxide NPs, silica NPs, etc., are being widely researched and used for anticancer therapy [9]. Each of these nanoparticles has its own unique features, which makes them a novel and efficient tool for anticancer therapy. Iron oxide NPs conjugated with anticancer drugs are used to make magneto-sensitive NPs for selective targeting using magnetic fields in cancer treatment [10]. Likewise, titanium dioxide NPs are used in photodynamic therapy used for cancer therapy. They are used as a replacement for photosensitizer, which is excited by radiation to induce Reactive oxygen species (ROS) generation and thus apoptosis [11, 12]. Cerium oxide NPs are used in radiation therapy for cancer treatment, in which they selectively kill irradiated cancer cells while posing no effects on the surrounding normal cells [13]. Zinc oxide NPs are also used for selective cytotoxicity towards cancer cells, where they show cytotoxicity by zinc-dependent protein activity disequilibrium and ROS induction [14]. Copper oxide NPs can be easily synthesized using plant extract such as Ficus religiosa [15] or Acalypha indica [16] and their synthesis methods are simple, non-toxic and eco-friendly [17]. The controllable pores of silica NPs make them a good carrier for drugs in anticancer therapy [18]. In addition, gold, silver and platinum NPs, known as precious metal or noble metal NPs, are also being used for cancer therapy as drug delivery and therapeutic agents [8]. The low reactive nature of these noble elements is advantageous for drug delivery purposes.

Among all these NPs, zinc oxide NPs are showing promising application and efficacy in cancer therapy due to their highly selective nature and potency towards cancer cells. This review aims to explore these unique properties of ZnO NPs, their role in the human body and their mechanism of cytotoxicity towards cancer cells.

## 2. ZnO Nanoparticles

Nanotechnology deals with controlling, modifying and fabricating materials, structures and devices with nanometre precision. It helps to understand the fundamental physics, chemistry, biology and technology of nanometre-scale objects [19]. ZnO nanoparticles are nano-sized particles of ZnO with a size less than 100 nm. They can be prepared by several different methods, such as solid, liquid (i.e., chemical) and gaseous. There are a variety of chemical methods, for example mechanochemical process, precipitation process, precipitation in the presence of surfactant, sol-gel method, solvo-thermal, hydrothermal, emulsion and micro-emulsion methods [20]. The chemical method is the most cost-effective, reliable and environmentally friendly and also provides flexibility for controlling the size and shape of synthesized nanoparticles. Nanoparticles with high surface area to volume ratio are preferred and, to make these types, stabilization of the nanoparticles is important. The synthesis of particles in nano size only is not fruitful in its application as these can again easily agglomerate into macro-sized particles. Therefore, they are stabilized by using surfactant, polymer molecules, or any organic molecules bound to the surface of nanoparticles, for example, Triton-X 100 or PEG. The main significance of nanoparticles is that the size reduction to nanoscale may lead to the development of new unique physicochemical, structural, electronic and magnetic properties of nanoparticles, which are not present in their macro or bulkier form [21]. These novel properties are mainly responsible for the unique and vast application of nanoparticles in the biological and medical field. ZnO nanoparticles now have a wide range of applications in cancer therapy, biosensing, drug/gene delivery, nanomachines that can act as biological mimetic, biomaterials for tissue engineering, shape-memory polymers such as molecular switches, etc. Owing to this wide application of ZnO nanoparticles, a variety of ZnO nanostructures have been synthesized, including nanoparticles, nanowires, nanorods, nanotubes, nanobelts and other complex morphologies [22].

### 2.1 ZnO nanoparticles: Promising for anticancer therapies

A ZnO nanoparticle, as a wide band-gap semiconductor, can readily absorb UV rays. Owing to this property, ZnO nanoparticles have a wide range of application, from electronic devices, cosmetics and facial products to biomedical application. ZnO nanoparticles are now being widely researched for their anticancerous properties. Some of the characteristic features of ZnO nanoparticles behind their surge in anticancer therapy are described below.

#### 2.1.1 Biocompatibility

ZnO nanoparticles show relatively high biocompatibility. Their bulkier form is generally recognized as safe (GRAS) by the FDA. Zinc is an important co-factor in various cellular mechanisms and plays an important role in maintaining cellular homeostasis; hence ZnO shows biocompatibility. The administered ZnO can be easily biodegraded or can take part in the active nutritional cycle of the body [23].

#### 2.1.2 Selectivity

ZnO nanoparticles have an inherent nature of showing selective cytotoxicity against cancerous cells in in vitro condition compared with other nanoparticles. They can be further surface engineered to show increased selective cytotoxicity [24].

#### 2.1.3 Easy synthesis

The synthesis process of ZnO nanoparticles is relatively easy, with a wide variety of methods. Owing to these different methods of synthesis, their size and size distribution can be easily controlled. Research has shown that the size of nanoparticles is directly proportional to the toxicity they show; in addition, size manipulation is significant for producing greater EPR effect to increase intra-tumour concentration of nanoparticles [25].

#### 2.1.4 Enhanced cytotoxicity

While extracellular ZnO shows biocompatibility, elevated levels of administered intracellular ZnO show enhanced cytotoxicity through zinc-mediated protein activity disequilibrium and oxidative stress [14]. ZnO nanoparticles have the unique ability to induce oxidative stress in cancer cells, which has been found to be one of the mechanisms of cytotoxicity of ZnO nanoparticles towards cancer cells. This property is due to the semiconductor nature of ZnO. ZnO induces ROS generation, leading to oxidative stress and eventually cell death when the anti-oxidative capacity of the cell is exceeded [26].

### 2.2 Key properties of nanoparticles

#### 2.2.1 Size

Size is one of the key properties of nanoparticles. A size range of 10-100 nm is considered good for biological application. The lower scale of this size range is based on the measurement of the sieving coefficient for the glomerular capillary wall, as the threshold for first pass elimination by the kidneys is estimated to be 10 nm in diameter. The upper scale of the size range is based on the result of various studies showing that, up to 100 nm, nanoparticles show better efficacy. Some research articles show that nanoparticles smaller than 10 nm diameter accumulate more efficiently and penetrate more deeply in tumours than their larger counterparts [25], but extensive research is necessary in this area as nanoparticles smaller than 10 nm may have a wide range of toxic issues in normal cells and the overall body. Nano size allows nanoparticles to enter inside the cell easily and manipulate the cellular function of the cell, leading to cytotoxicity. Nanoparticles can interact with biological molecules and manipulate various cellular cycles, disrupting cellular homeostasis and inducing apoptosis, due to their small size, which cannot be checked by the plasma membrane.

Tissue resident macrophage in the liver and spleen rapidly clears most particles entering into blood vessels. A blood protein called ‘opsonins’ is adsorbed in any foreign particles entering blood and macrophage targets these adsorbed opsonins. Research has suggested that size is related to blood circulation time of particles. The smaller the size, the more the blood circulation time. In addition, small size, i.e., less than 10 nm, shows increased cytotoxicity by means of increased diffusion through cytoplasm. These ultra-sized particles even enter the nucleus and show toxicity through nucleus aberration [25].

#### 2.2.2 High surface area to volume ratio

Nanoparticles have high surface area to volume ratio due to their nano size. This property of nanoparticles allows increased surface contact and increased reactivity and solubility and other wide ranges of application. Various ligand and targeting molecules can be conjugated in the surface of nanoparticles, which is an important aspect of nanoparticle-mediated drug delivery.

The surface charge of a nanoparticle also has an important effect on its fate. High surface charges lead to increased macrophage scavenging activity, resulting in rapid clearance of nanoparticles from blood vessels. Similarly, surface charge should be maintained in such a way that it should have minimal self-self and self-nonself interaction while displaying selectivity towards tumour cells. The ultimate fate of nanoparticles depends upon their interaction with their surrounding environment, which ultimately depends upon the size and surface properties of nanoparticles [27]. The other properties of nanoparticles also include chemical composition of high purity, crystallinity, and quantum effects that can affect chemical reactivity.

## 3. Zinc and Cancer

### 3.1 Zinc and its role in the human body

Zinc is a silver-grey-coloured transition metal with oxidation state +2, having five stable isotopes. It is the most important and abundant trace element in the body after iron. The total body zinc content has been estimated to be 30 mM (2g). It is required in the human diet in trace quantities, which is approximately 15 mg Zn per day [28]. It is present in all body tissues and fluid. Zinc plays a major role in the immune system, affecting a number of aspects of humoral and cellular immunity. It helps to maintain cell and organ integrity by stabilizing the molecular components of membrane and cellular components. Zinc has a role in regulating a large number of enzymes present in the body, which later participate in synthesis and degradation of carbohydrates, lipids, proteins and nucleic acid, as well as in metabolism of other micronutrients, thus playing a crucial role in maintaining proper body condition and homeostasis. It also plays a role in genetic expression by regulating polynucleotide transcription. Concentration-dependent absorption of zinc occurs throughout the small intestine, whereas zinc is lost from the body through the kidneys, skin and intestines [29].

### 3.2 Zinc deficiency leads to initiation and progression of cancer

Zinc is the co-factor of over 300 mammalian enzymes and plays a vital role in host defence against the initiation and progression of cancer [30]. The tumour suppressor p53 gene and caspase enzyme help to check cells regularly and prevent them from becoming cancerous. If a cell shows any kind of malignancy, a DNA repair mechanism is activated to repair the altered DNA. If this mechanism fails to repair the DNA, then the cell undergoes ‘programmed cell death’, known as apoptosis, to prevent the altered cell from dividing, which may later develop in the cancerous cell. In one way or another, zinc is involved in all these processes of protecting cells against cancer.

The exact mechanism for inducing apoptosis is not clear, but mutation or damage to DNA appears to play a major role in triggering activation of the p53 gene, which leads to apoptosis [31]. The specific DNA-binding domain of p53 contains a complex tertiary structure that is stabilized by zinc [32]. Thus, zinc plays a major role in maintaining the activity of tumour suppressor gene p53, which regulates apoptosis activity of cells. Similarly, zinc also plays a crucial role in the activation of the caspase-6 enzyme, a major enzyme responsible for apoptosis. Caspase-6 is the most sensitive apoptosis-related molecular target of zinc. It is responsible for the activation of caspase-3 and other enzymes that are responsible for nuclear membrane dissolution leading to cell death [33]. Several zinc channels maintain a crucial balance between life and cell death, controlling the intracellular zinc movements and free amount of the metal. While low concentration of zinc can lead to the initiation and progression of cancer, high concentration of zinc also has a deleterious effect on health. An excess amount of zinc that exceeds the capacity of the zinc homeostasis system can lead to a breakdown of the zinc transporting system of the plasma membrane, resulting in increased intracellular zinc concentration, which ultimately activates apoptosis, causing cell death [34]. The deleterious effect of excess zinc is discussed in detail later in the section “Mechanism of cytotoxicity of ZnO NPs”.

Zinc plays an important role in response to oxidative stress, DNA replication, DNA damage repair, cell cycle progression and apoptosis; hence a deficiency of zinc leads to disruption of crucial homeostasis in cells. A decrease in cellular zinc alone causes DNA damage and impairs the DNA damage response mechanism, resulting in a loss of DNA integrity and potential for increased cancer risk. It has been observed that low intracellular zinc induces oxidative DNA damage, disrupts p53 and affects DNA repair in rat glioma cell lines [35]. Likewise, an increase in serum copper/zinc ratios in patients with cancers of the lung, breast, gastrointestinal tract and gynaecological malignancy has been observed [36]. In addition, significantly lower than normal tissue zinc concentration has been demonstrated in oesophagus cancer [37]. Similarly, evidence suggests that zinc accumulation is an important factor in the progression and development of prostate cancer, often characterized by low zinc concentration in malignant prostate cells as a result of downregulation of ZIP 1, a zinc transporter protein [38].

All these research findings suggest that cancerous cells are characterized by a decrease in zinc concentration or altered zinc concentration. A deficiency of zinc can make cells unstable and prone to cancer. It is an important factor in the development and progression of malignancy. Therefore, zinc-mediated cancer chemoprevention could be efficacious in the prevention and treatment of several cancers.

## 4. Interaction of Nanoparticles with Biological Components

Nanoparticles act on a nano level. Therefore, understanding the interaction of nanoparticles with the cell surface is important in understanding the mechanism of their action and manipulating it for medical and biological application. Nano-bio interface is a dynamic physicochemical interaction between the nanomaterial surface and the surface of biological components, which deals with the kinetic and thermodynamic exchanges between the interface [39]. It includes the interaction between the nanoparticles' surfaces, the solid-liquid interface and the solid-liquid interface contact zone with the biological membrane.

Besides the nano-bio interface, another important interaction that defines the role of nanoparticles is the interaction between nanoparticles themselves. Various forces, such as van der Waals forces, electrostatic force, solvation, solvophobic and depletion forces act on these interactions, which affect the rate of agglomeration of nanoparticles in media [40]. An understanding of this interaction is important for the proper dispersal of nanoparticles in the media with minimal agglomeration. Furthermore, the use of optimal surfactants and polymers to stabilize nanoparticles can be found by studying these interactions.

Van der Waals and depletion forces act as attractive forces, whereas electrostatic force acts as a repulsive force. Van der Waals forces arise from changes in the dipole moment of electrons, which induce a dipole moment in the adjacent atoms. It arises from the quantum mechanical dance of electrons [41]. These different forces play an important role in the adhesive interaction of nanoparticles in the cell surface and their passive uptake inside the cell [42, 43]. Their passive uptake inside the cell. This adhesive interaction to the cell surface is favored by the optimizable surface area of nanoparticles according to the cell surface receptor, and the comparative size of nanoparticles to that of ligands/biomolecules, leading to adhesive interaction and passive uptake inside the cell, boycotting the phagocytic process [44, 45]. With this passive uptake, nanoparticles can directly interact with cytoplasm proteins and cell organelles, leading to increased cytotoxicity. They can localize anywhere inside the cell including the outer membrane, cytoplasm, lipid vesicles, mitochondria, nuclear membrane, nucleus, DNA, etc., damaging these cell organelles and ultimately leading to cell death [46].

### 4.1 Protein corona determines the fate of nanoparticles

The fate of a nanoparticle, i.e., the biological area that it will target, is known to be dependent upon the nature of the particle itself, i.e., its hydrophobicity, size, radius of curvature, charge, coatings, etc. These factors influence the protein coating that is formed around nanoparticles when it enters a biological fluid. This coated-protein structure of nanoparticles is called a protein corona. The coated protein may undergo conformational change, leading to the exposure of new epitopes and altered function. Protein corona interacts with certain biological membranes while showing no interaction with certain other biological membranes. It shows specificity, thus determining the fate of nanoparticles. Protein binding to nanoparticles also helps to increase the dissolution rate of nanoparticles [47, 48].

**Figure 1.**
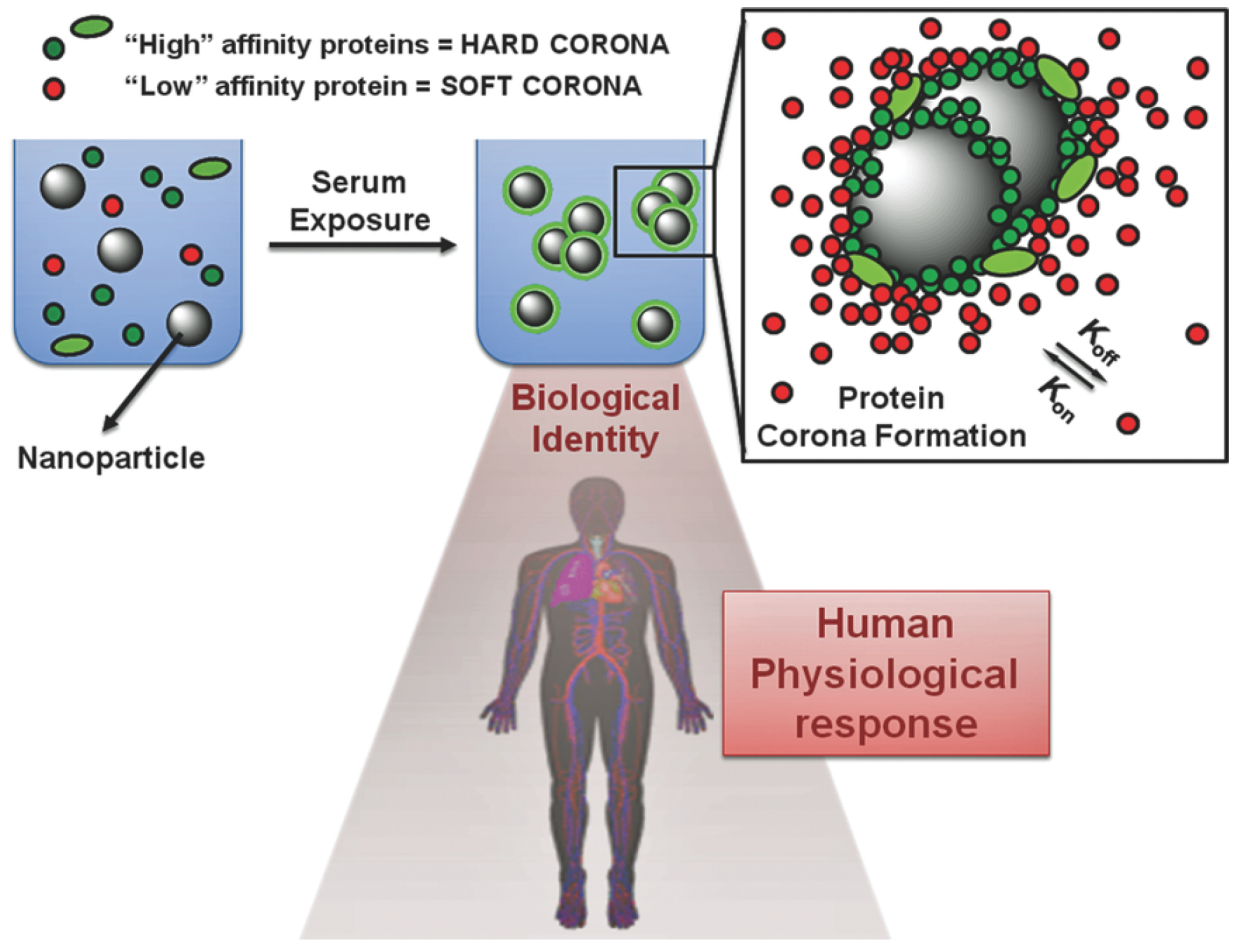
A pictorial representation of the formation of a protein corona when nanoparticles are exposed to serum. This figure represents the interaction of nanoparticles once they are injected inside the body. Biomolecules, especially protein, form a thin layer of segment around the surface of nanoparticles, known as a protein corona. High-affinity proteins (green) bind tightly with the nanoparticle surface, forming a first layer of hard corona followed by the reversible adsorption of low-affinity protein forming a soft corona. (Figure taken from research article “Verderio P, Avvakumova S, Alessio G, Bellini M, Colombo M, Galbiati E, et al. Delivering Colloidal Nanoparticles to Mammalian Cells: A Nano–Bio Interface Perspective. Advanced Healthcare Materials. 2014;3[7]:957-76.” Licence granted by John Wiley and Sons under partnership with copyright clearance centre for the reuse of above figure. Licence Number: 3850911134439).

As depicted in Figure 1, a protein corona forms once nanoparticles are injected inside the body, where it interacts with serum biomolecules, especially protein. High-affinity proteins bind tightly with the nanoparticle surface, forming a first layer of hard corona followed by the reversible adsorption of low-affinity protein, forming an additional layer of soft corona [49]. The nature of nanoparticles, such as size, charge, hydrophobicity, etc., contributes to the formation of the corona. Various interactions take place between the corona and the surrounding environment and the corona dynamically changes. These interactions include steric hindrance, protein binding capability, available surface area, binding interaction and characteristic protein attachment/detachment. Steric hindrance prevents binding of the corona to other particles. The available surface area, surface coverage and angle of curvature determine the adsorption profile of nanoparticles. Binding interaction releases surface-free energy, leading to surface reconstruction of the corona. Competitive binding interaction depends upon protein composition and body fluid composition. Characteristic protein attachment/detachment depends upon the material type and protein characteristic. All these interactions help to change the corona dynamically. The corona changes when particles translocate from one biological compartment to another. In the process of formation of the protein corona, when the protein interacts with nanoparticles, they undergo potential changes in their structure and function. Basically, protein conformational change takes place, leading to protein fibrillation, loss of enzymatic activity, protein crowding, etc., and surface opsonization of the corona takes place, allowing additional nano-bio interaction.

Thus, the nanoparticle-protein corona interaction is important for understanding the surface properties, charge, resistance to aggregation, hydrodynamic size, specificity and biological targets of nanoparticles [48], and these factors play an important role in the cellular uptake and subcellular localization of nanoparticles [50].

## 5. Mechanism of Cytotoxicity

The basic mechanism behind the cytotoxicity of ZnO NPs is the intracellular release of dissolved zinc ions, followed by ROS induction. This event causes zinc-mediated protein activity disequilibrium and oxidative stress, eventually killing the cell. Soluble extracellular zinc shows very little cytotoxicity. Recent research shows that extracellular soluble zinc, when exposed to cell culture and media, forms poorly soluble amorphous zinc-carbonate phosphate precipitates (phosphate from media). This precipitate is supposed to protect the cell from the cytotoxicity of zinc [51]. On the other hand, with the release of soluble zinc ions inside the cell, a cascade of pathways interrelated to each other takes place, which is responsible for the cytotoxic response of the ZnO nanoparticles. These events can be described in three major topics, as follows:

### 5.1 Zinc-mediated protein activity disequilibrium

Zinc is the one of the major trace elements found in the human body and is maintained in a definite concentration inside a cell [52]. Alteration in this concentration of zinc in the cell may cause severe problems in various cellular processes, as zinc is the co-factor of more than 300 mammalian enzyme [30]. With the application of ZnO NPs and the intracellular release of zinc ions, the concentration of zinc in the cell rises from normal level, resulting in zinc-mediated protein activity disequilibrium. This affects a wide range of crucial cellular processes, including DNA replication, DNA damage repair, apoptosis, oxidative stress, electron transport chain, cellular homeostasis, etc., rendering cytotoxicity towards the cell [14].

**Figure 2.**
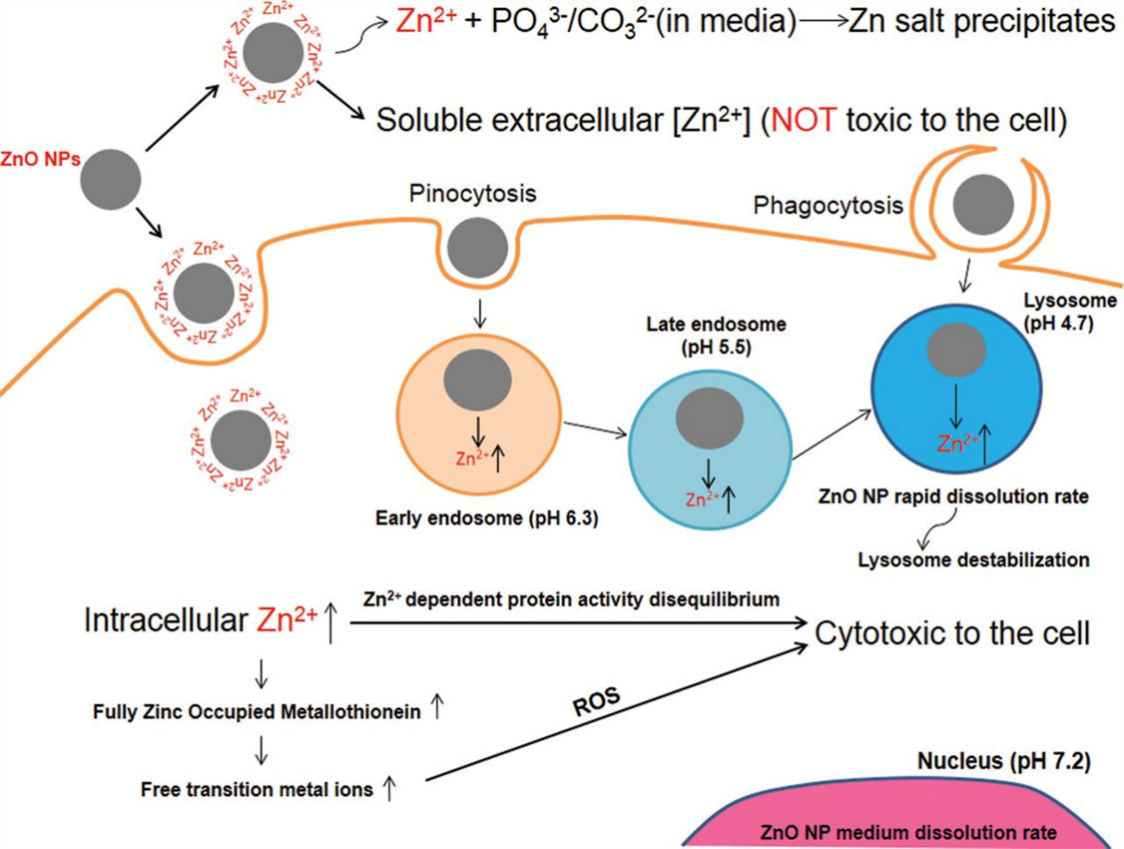
A schematic representation of the mechanism of cytotoxicity of a nanoparticle. (Figure taken from open source article “Shen C, James SA, de Jonge MD, Turney TW, Wright PF, Feltis BN. Relating cytotoxicity, zinc ions, and reactive oxygen in ZnO nanoparticle-exposed human immune cells. Toxicol Sci. 2013;136[1]:120-30”. Licence granted by Oxford University Press under partnership with copyright clearance centre for the reuse of above figure. Licence Number: 3850790022302).

As illustrated in Figure 2 above, first of all ZnO nanoparticles are taken up by the cell through endocytosis. Some nanoparticles simply enter the cell, whereas some enter through pinocytosis and phagocytosis bounded by endosomes and lysosomes. As pH decreases, the ZnO nanoparticles' dissolution rate increases rapidly, causing lysosome destabilization [53]. The pH of early endosome is relatively low, i.e., 6.3, which favours the release of soluble zinc ions. It further decreases to pH 5.5 at late endosome and pH 4.7 in lysosome, where a rapid dissolution rate of ZnO NPs is observed, causing lysosome destabilization. This suggests that, for the release of zinc ions, low pH is necessary and thus the release of zinc ions in blood or extracellular fluid, which has a normal pH of 7, is not favourable [14]. This process leads to an increased release of soluble zinc ions inside the cell. The increase in intracellular zinc concentration leads to zinc-dependent protein activity disequilibrium, thus resulting in cytotoxicity to the cell. The increase in soluble zinc ions also increases ROS concentration, leading to cytotoxicity of cells through oxidative stress [54].

### 5.2 ROS production and oxidative stress

ROS, a reactive species of molecular oxygen, is produced inside the cell during various cellular processes, including mitochondrial respiration, inflammatory response, microsome activity, peroxisome activity, etc. It acts as a biomolecule and plays an important role in cell signalling and homeostasis. Exogenously, ROS is induced in response to various stimuli including nanomaterials [55]. ROS is induced by ZnO NPs in two ways. One is due to the pro-inflammatory response of the cell against nanoparticles [56] and the other is due to the characteristic surface property of ZnO NPs that makes them a redox reaction system producing ROS [26, 57].

The ability to induce oxidative stress through ROS generation by ZnO nanoparticles is due to its semiconductor properties. ZnO is a wide band-gap semiconductor with direct band-gap of 3.37 eV and large excitation binding energy of 60 MeV [58]. Being a semiconductor, it does not have a continuum band of electronic states like other metal. The valence band and conduction band are separated by a considerable large energy gap, i.e., 3.37 eV. Normally, UV light is required to elucidate electrons (e-) from the valence band to reach the conduction band, leaving holes (h+) in the valence band. Conduction of electricity takes place by the movement of free electrons in the valence band. However, in the case of the nano-sized ZnO nanoparticles, electrons also jump to the conduction band in the absence of UV irradiation [59]. Electrons and holes often recombine quickly but in the case of nanoparticles, they move to the nanoparticle surface, where they react with the adsorbed species. This results in an increased number of electrons and holes in the nanoparticle surface. This peculiar characteristic of nanoparticles may be due to the crystal defect in nanoparticles, due to their nano size.

Holes (h+) act as a powerful oxidant, breaking water molecules into hydrogen and hydroxyl ions. Similarly, electrons act as a powerful reducer, reacting with adsorbed and dissolved oxygen molecules, generating superoxide radical anions (O_2_^−^). These superoxide radical anions further react with hydrogen ions, producing HO_2_^−^ radicals, which further react to create H_2_O_2_. All these radicals are a highly reactive oxygen species known as ROS, which acts as a strong oxidizing agent. Accumulation of these species in great amounts leads to a misbalance in the oxidative-reductive homeostasis of the cell, leading to oxidative stress, which is very harmful to the cell and eventually causes cell death. Thus, holes and electrons in ZnO nanoparticles act as a redox reaction system, producing a reactive oxygen species and thus increasing oxidative stress in the cell [26].

### 5.3 DNA damage and apoptosis

With elevated levels of ROS and oxidative stress, ZnO NPs show a deleterious effect on the lipid, protein and nucleic acid of the cell [60]. Elevated ROS can cause membrane damage through lipid peroxidation and protein denaturation, resulting in cell death by necrosis and DNA damage, resulting in cell death by apoptosis [55]. DNA damage mainly occurs by DNA strand breaks and DNA protein cross-links [61, 62]. Highly reactive species of ROS can react with components of DNA, altering DNA composition and bringing mutation in DNA. OH radical, a highly reactive species of oxygen, causes single stranded breakage in DNA via the formation of 8-hydroxy-2-deoxyguanosine (8-OHdG) DNA adduct [63]. These DNA breakages and crosslinks damage DNA, leading to the activation of a mitochondrial apoptotic pathway, eventually causing cell death by apoptosis [31]. Apoptosis, a programmed cell death, is believed to be the major mechanism of cell death in this cytotoxic response of ZnO NPs [64, 65].

**Figure 3.**
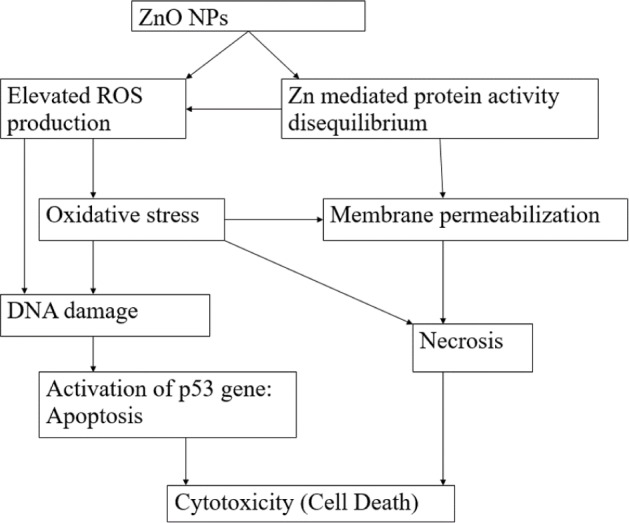
A schematic representation of the overall cytotoxicity of ZnO nanoparticles, leading to cell death

Figure 3 shows the overall mechanism of cytotoxicity of ZnO NPs. As represented in the figure, zinc-dependent protein activity disequilibrium and elevated ROS production leads to cytotoxicity. Research into ROS has shown that the use of antioxidants and ROS quenchers did not significantly decrease the cytotoxicity of ZnO NPs [14, 51, 54]. This suggests that ROS production might not be the main mechanism of cytotoxicity of ZnO NPs, but rather the cytotoxic response instead. Although ROS is also produced by the interaction of ZnO with the cell, the main source of ROS production may be due to the cytotoxic response of zinc-dependent protein activity disequilibrium, such as the permeabilization of mitochondria releasing huge amounts of ROS in the cell. This leads to the conclusion that the probable main mechanism of cytotoxicity of ZnO NPs is zinc-dependent protein activity disequilibrium as a result of the increased dissolution of free zinc ions inside the cell [14, 51, 54].

## 6. Selective Cytotoxicity Towards Cancer Cells

Many in vitro studies have proved that ZnO NPs show selective cytotoxicity towards cancer cells [66–70]. Hanley suggested that they show 28-35 times selective toxicity towards cancer cells compared with that of normal cells [24]. This selective cytotoxicity in cancer cells in in vitro condition can also be further exploited in the in vivo condition by selectively targeting ZnO nanoparticles towards cancer cells. ZnO NPs selectively kill cancer cells by inferring selective localization and selective cytotoxicity towards them.

### 6.1 Selective localization through EPR and electrostatic interaction

One of the characteristics of nanoparticles is that they show enhanced permeation and retention effect (EPR) in tumour cells because of their small size and surface properties. Due to the rapid and uncontrolled growth of tumours, tumour cells lack properly developed blood and lymphatic vessels. Cell-cell connections in a tumour cell are also weak due to an improperly developed tight junction. Blood vessels running through a tumour have pores ranging in size from 100 nm to 1 micrometre. Owing to these properties of cancer cells, nanoparticles can easily diffuse through the blood vessels towards a tumour cell, thus showing enhanced permeation selectively toward tumor cell. This process of movement of nanomaterials from blood to the tumour bulk is called extravasation. The extravasated fluids and particles are quickly carried away by the flow of interstitial fluid surrounding the cell, known as lymph in healthy tissue. However, since a tumour cell also has an improper lymphatic system, they are not carried away from the tumour tissue, immediately allowing enhanced retention time for nanoparticles in tumour tissues. The enhanced retention time allows enhanced diffusion of particles inside the tumour cell. Thus, the enhanced permeation and retention effect helps nanoparticles to be localized specifically in the tumour cell specifically and to act upon it [26, 27].

The electrostatic characteristic of ZnO nanoparticles is also useful for anticancer therapy as a selective targeting purpose. ZnO has a neutral hydroxyl group attached to its surface, which plays an important role in its surface charge behaviour. At high pH, ZnO exists as ZnO^−^ due to the transfer of adsorbed protons from its surface towards aqueous solution. At low pH (acidic condition), ZnO exists as ZnOH_2_^+^ due to the transfer of protons from the aqueous environment towards its surface. The isoelectric pH of ZnO nanoparticles is 9-10. Thus, ZnO nanoparticles exhibit positive charge under physiological conditions such as blood or tissue fluid (which has pH 7), etc. [26, 71]. On the other hand, cancerous cells usually have high concentration of (negatively charged) anionic phospholipids on their outer membrane [72]. This leads to an electrostatic attraction between ZnO nanoparticles and cancerous cells, thereby promoting selective localization, cellular uptake, phagocytosis and finally cytotoxicity. Selective localization of NPs can be further enhanced by proper surface engineering and modification.

### 6.2 Selective cytotoxicity through ROS

While many studies have demonstrated that ZnO NPs show selective cytotoxicity towards cancer cells, the exact mechanism of the selectivity is still unclear. ROS may provide a possible explanation for the selective cytotoxic response of ZnO NPs towards proliferating cells. It has been observed that ROS generation is relatively greater in cancer cells than in normal cells after ZnO NP treatment [66]. ROS and various signalling molecules are generally found in greater amount in rapidly proliferating cells such as cancer cells, owing to their faster metabolism rate compared with normal cells [73]. When ZnO NP treatment is given to cancer cells, then ZnO nanoparticles, being a redox reaction system in themselves, may react with the increased amount of chemical species and signalling molecules around them, producing even more ROS, resulting in huge oxidative stress in the cell and eventually killing the cell. While ZnO NP treatment also generates ROS in normal cells, the generation is relatively low compared with cancer cells, as initially they have less ROS and fewer signalling molecules that can be converted into more reactive species. Hence, the oxidative stress produced may not be enough to kill the cell and thus it shows a relatively lower cytotoxic response. This may, therefore, be the possible mechanism behind the selective cytotoxicity of ZnO NPs in proliferating cells, including cancer cells.

## 7. Conclusion

Nanoparticles, with their unique properties, are showing increasing application in cancer research and therapy. With their selective targeting property and usefulness as a carrier agent, ZnO NPs can be good substitutes for traditional cancer therapy. This review has mainly focused on ZnO NPs, the relation between zinc and cancer, zinc's role in the human body and the probable mechanism surrounding ZnO NPs with the biology of the human body, leading to its selective localization and cytotoxicity towards cancer cells. While ZnO NPs induce cytotoxicity towards cancer cells through oxidative stress via ROS generation, this may not be the main mechanism of cytotoxicity; rather, the response of zinc-mediated protein activity disequilibrium as a result of high levels of intracellular zinc ions is a more likely cause.

Nanoparticles in medicine are a new and emerging topic of interest for researchers. With all their promising characteristics, the in vivo application of nanoparticles is still rare and there is currently a serious lack of in vivo research into nanoparticles. Hence, a much better collaboration between clinicians, biologists and material scientists is required for the in-depth understanding of cancer biology and intelligent design of NPs for their better clinical use. A dynamic collaboration could lead to the development of smart NPs that show superior accuracy of selectivity and toxicity towards cancer cells while causing no harm to normal cells. This is in fact an achievable aim, considering the highly promising characteristics of ZnO NPs and their inherent nature of selectivity and toxicity towards cancer cells, making them unequivocally a key tool for next-generation cancer treatment.

## 8. Compliance with Ethical Research Standards

The authors declares no conflict of interest. All the works reported in this review article was performed in accordance with principle of ethical research that complies with all relevant legislation.
